# High-density lipoprotein is inversely associated with psychiatric symptoms across diagnoses in patients with general psychiatric disorders

**DOI:** 10.3389/fpsyt.2025.1634920

**Published:** 2025-09-05

**Authors:** Hedda Soloey-Nilsen, Kristin Nygaard-Odeh, Magnhild Gangsoey Kristiansen, Erling Inge Kvig, Ole Lars Brekke, Tom Eirik Mollnes, Michael Berk, Solveig Klaebo Reitan

**Affiliations:** ^1^ Department of Mental Health and Addiction Medicine, Nordland Hospital Trust, Bodoe, Norway; ^2^ Institute of Clinical Medicine, University of Tromsoe (UIT) The Arctic University of Norway, Tromsoe, Norway; ^3^ Research Laboratory, Nordland Hospital Trust, Bodoe, Norway; ^4^ Department of Immunology, Oslo University Hospital and University of Oslo, Oslo, Norway; ^5^ Centre of Molecular Inflammation Research, Norwegian University of Science and Technology, Trondheim, Norway; ^6^ School of Medicine, Barwon Health, the Institute for Mental and Physical Health and Clinical Translation (IMPACT), Deakin University, Geelong, VIC, Australia; ^7^ Orygen, The National Centre of Excellence in Youth Mental Health, Centre for Youth Mental Health, Florey Institute for Neuroscience and Mental Health, Melbourne, VIC, Australia; ^8^ Department of Psychiatry, The University of Melbourne, Melbourne, VIC, Australia; ^9^ Department of Mental Health, Faculty of Medicine and Health Sciences, Norwegian University of Science and Technology, Trondheim, Norway; ^10^ St Olavs Hospital, Nidelv Community Center of Mental Health, Trondheim, Norway

**Keywords:** HDL, LDL, lipids, mental health, neurosciences, psychiatry, symptoms, TG

## Abstract

**Background:**

Lipids are essential in cell structure and function in all parts of the body including the brain. Thus, lipids are of obvious relevance in psychiatric disorders. While the role of lipids in pathophysiological processes in cardiovascular disorders are widely known, the role of lipids in function and pathophysiology of mental processes are far less established. This study aimed to explore serum lipid levels and their association in a clinical cohort with general psychiatric symptoms.

**Methods:**

A transdiagnostic sample of 132 patients was recruited from a general open psychiatric ward to this cross-sectional naturalistic study. Psychiatric symptoms were assessed using the Symptom Checklist-90-Revised (SCL-90-R). Serum levels of triglycerides (TG), low-density lipoprotein (LDL) and high-density lipoprotein (HDL) were measured, and multiple linear regression analysis were performed to investigate associations with symptom clusters from SCL-90-R.

**Results:**

After correcting for the most common confounding factors, HDL was negatively associated with intensity of phobic anxiety (*p*=0.021), paranoid ideation (*p*=0.041), anger hostility (*p*=0.033) and interpersonal sensitivity (*p*=0.003) symptom clusters. No relations were found between TG, LDL and symptom clusters.

**Conclusion:**

HDL was significant inversely associated with several general psychiatric symptoms. This result indicates a role for lipids in the pathophysiology of psychiatric disorders and suggests a mechanism for the increased cardiovascular risk across psychiatric diagnoses.

## Background

Psychiatric disorders remain among the top ten leading causes of disease burden worldwide (1). Despite the fact that there has been substantial efforts to develop new prevention and treatment strategies as well as abundant search for novel mechanisms of action, there are still considerable unmet needs (2). A particular unmet need is the very high rate of cardiovascular disease across psychiatric disorders accounting for the bulk of the premature mortality in this group (3). This supports closer follow up of lipid levels.

After adipose tissue, the brain has the next highest lipid content and brain lipids constitute 50% of the dry weight of the brain (4). Lipids are essential to brain function, and any deviation in structure and level of brain lipids have the potential to cause disease to the central nervous system (5). Increasing attention has been paid to the role of lipids in psychiatric suffering. Lipids are essential in cellular structure and function owing to their role in cellular membrane formation and organization, transportation, signal transduction and neuroplasticity (6, 7). Lipids and lipid soluble molecules in blood enter the brain. Serum concentrations of lipids are a useful method to measure lipids and their associations, as an indirect assessment of brain lipid status (8). Triglycerides and cholesterol are among the most important lipids in the brain (9, 10).

There is a putative role of dyslipidaemia in pathogeneses of psychiatric disorders. Accordingly, correction of dyslipidaemia in treatment of these disorders and a role of measuring levels of peripheral lipids in assessing type and severity of psychiatric illness are potentially clinically useful (11, 12). Also, statins may have transdiagnostic value in psychiatric disorders, with evidence in psychosis and depression (13, 14). However, to apply this into clinical practice, more accurate knowledge on the role of different lipids in relation to psychiatric suffering is needed.

Previous research has suggested high levels of TG and low levels of LDL and HDL may be associated with higher risk of psychiatric illness (15, 16). Reduced serum HDL levels have been linked to major depression, and suicidal behaviour (17, 18). A positive association is found in some studies between TG, LDL and depression (19), though this is not consistent (20). Other psychiatric diagnoses are found to be inversely associated with HDL levels; mood disorders, schizophrenia and anxiety disorders (21, 22).

There are also reports on relations between lipids and psychiatric symptoms including violent behaviour (23), depression and anhedonia (24). Across some studies anxiety symptomatology was accompanied by an increase in TG (8). However, potentially mediating factors or heterogeneity of symptoms is not always considered (25). Severe psychiatric disorders are to an increasing extent not merely looked upon as a disease of the mind, but as a systemic disorder (26). As such, these involves nervous, immune and endocrine as well as metabolic systems (27). This supports a closer follow-up of lipids also for patients with general psychiatric disorders. Further investigations of this link could pave the way for more personalized and symptom-oriented treatment for psychiatric disorders. As symptoms often overlap and co-occur in psychiatric diagnoses a further symptom-based search across diagnostic boundaries may be of relevance (28).

The aim of the present study was to explore associations between serum TG, LDL, HDL and psychiatric symptoms in a sample of patients admitted to an open psychiatric inpatient ward.

## Material and methods

### Study design, recruitment and participants

In this cross-sectional naturalistic study 132 patients were enrolled from an open inpatient psychiatric ward, at the Department of Mental Health and Addiction medicine, Nordland Hospital Trust, Bodoe, Norway. Patients were not acutely ill or with severe mental illness, their main complaint for admission to this ward was common mental disorders (depression, anxiety disorders etc). The cohort has been previously described (29).

Patients aged 18 years and above were recruited over a 4-year period from February 2014 to February 2018. Patients were referred to this ward from the hospital’s outpatient services and from general practitioners. We do not have information about illness duration. Patients not giving their consent and/or not understanding the Norwegian language or who were otherwise unable to give informed consent were not enrolled (29).

### Ethics

A research nurse informed eligible patients about the study and written informed consent was obtained by a doctor administering the clinical assessments. The study was approved by the Regional Ethics Committee (notification 2015/1809/REK Nord) and was performed in accordance with the Helsinki declaration.

### Data collection and assessment of psychiatric symptoms

Age and gender were derived from the hospital`s personal identification data. Weight, height and smoking habits were obtained from the patients by history and examination. Body mass index (BMI) was calculated from the formula BMI= weight (kg)/height (m)^2^. All patients were assessed by the first author (HSN) upon consultation approximately one week after admission to the ward. At assessment the Symptom Checklist-90-Revised (SCL-90-R) was administered. SCL-90 is a validated 90 item rating scale for monitoring multiple symptoms and symptom clusters experienced by the patient over the last week. SCL-90-R is psychometrically valid for measuring psychopathological status, measuring change in outcome studies, or screening for mental disorders. Each of the 90 items is rated on a five-point scale, ranging from, not at all (0) to extremely (4) (30). The 90 single items are often grouped as primary dimensions or clusters: Depression, somatization, obsessive-compulsive, interpersonal sensitivity, anxiety, anger-hostility, phobic-anxiety, paranoid ideation, and psychoticism. All symptom clusters are measured with raw scores in our study. The global severity index (GSI) provides measures of overall distress (29).

### Blood sampling and biological measures

Blood samples from the patients were withdrawn by trained technicians in the morning the day after the clinical assessment between 08:00–10:00 a.m., after approximately 12 hours of fasting and rest. Biochemical measures were performed at the Department of Laboratory Medicine, Nordland Hospital Trust. For measurements of serum lipids; including TG, LDL, HDL, blood was withdrawn in Vacuette gel-tubes, and left for 30 minutes on ice before centrifugation at 2200 rpm for 15min. HbA1c (%) in EDTA whole blood was analysed using a Tosoh G8 high-performance liquid chromatography instrument (Tosoh Bioscience, Inc., San Francisco, CA). Serum TG, LDL, HDL and glucose were analysed on an ADVIA^®^ 1800 instrument (Siemens Medical Solutions Diagnostics, Japan). Blood sampling have been previously described (29).

### Statistical analysis

Multiple linear regression analysis was performed using TG, LDL and HDL as dependent variables and symptom clusters as well as possible confounding factors as age, gender, BMI and smoking as independent variables. All analyses were two-tailed, and test for normality were performed with Kolmogorov-Smirnov test and Q-Q plots, and HDL, TG and LDL were close to normal distributed. No-collinearity problem was detected, evaluated with residual analysis and testing with variance inflation factor below 10. For all analyses the IBM-SPSS version 28.0 was used, and the statistical significance was set at *p* < 0.05.

## Results

### Demographics

Demographic characteristics of the 132 study participants have been previously described and are presented in **Table 1**; 84 were women and 48 men with mean age 37 years (29).

**Table 1:** Demographic characteristics of study participants.

Results under demographics	N	%		
Gender (male)	47	36		
Smoking	60	46		
	Min	Max	Mean	SD
BMI	15	55	28	7
Age (years)	18	78	37	14

BMI, Body mass index; SD, standard deviation.

### Biochemical measures

Descriptive statistics of lipids presented in **Table 2**. The mean TG level was 1.6 mmol/L, SD 1.1, mean LDL level 3.2 mmol/L, SD 1.0, mean HDL level was 1.3 mmol/L, SD 0.4. All mean values were within the reference window used by the laboratory; reference interval TG; 0.5–2.6 mmol/L, LDL 1.2–5.2 mmol/L, HDL 0.8–2.7 mmol/L.

**Table 2:** Descriptive statistics of serum lipid levels.

Lipid	Min	Max	Mean	SD
Triglycerides mmol/L	0.4	8.2	1.6	1.1
LDL mmol/L	1.0	5.9	3.2	0.9
HDL mmol/L	0.7	2.7	1.3	0.4

HDL, High-density lipoprotein; LDL, Low-density lipoprotein.

The mean HbA1c level was 5.3% (34 mmol/mol) and the mean fasting blood glucose level was 5.4 mmol/L. When 10 outliers of HbA1c > 6% (42 mmol/mol) and 9 outliers of fasting blood glucose > 7.0 mmol/L were removed, the mean HbA1c was 5.2% (33 mmol/mol) and mean fasting blood glucose was 5.2 mmol/L. Outliers of HbA1c and fasting blood glucose that could indicate diabetes were removed in order to exclude patients with possible diabetes since prediabetes and diabetes mellitus is associated with several changes in lipid levels (31).

### Psychometrics

The highest scores were on depression, obsessive-compulsive, somatization, and anxiety symptom clusters in the SCL-90-R (29) (**Table 3**).

**Table 3:** Range of symptom clusters, raw scores from SCL-90-R.

Results under psychometrics	Mean	Minimum	Maximum
Depression	27	0	46
Somatization	19	0	46
Anxiety	17	0	37
Phobic anxiety	10	0	25
Paranoid ideation	7	0	20
Anger hostility	4	0	21
Psychotisism	9	0	30
Obsessive compulsive	20	0	38
Interpersonal sensitivity	16	0	35
Global severity index	12	0	22

### Lipids in relation to psychiatric symptom clusters

There were no significant associations between fasting TG and LDL and any symptom clusters (all *p >*0.05). A negative association was found between HDL and the following symptom clusters: phobic anxiety (*p=* 0.021), paranoid ideation (*p=* 0.041), anger hostility (*p*=0.033), and interpersonal sensitivity (*p=* 0.003). (**Table 4**). The result did not change upon correction for potential confounders age, gender, smoking and BMI. When outliers of HbA1c and fasting blood glucose were removed to exclude diabetes, the result did not change.

**Table 4:** Multiple linear regression analysis of HDL and symptom clusters.

Results under lipids in relation to psychiatric symptom clusters	Standardized beta coefficient	*P*-value*	95% CI
Depression	−0.1	0.326	(−0.01 to 0.03)
Somatization	−0.1	0.119	(−0.01 to 0.00)
Anxiety	−0.2	0.073	(−0.01 to 0.00)
Phobic anxiety	−0.2	**0.021**	(−0.02 to −0.01)
Paranoid ideation	−0.2	**0.041**	(−0.03 to −0.01)
Anger hostility	−0.2	**0.033**	(−0.04 to −0.02)
Psychotisism	−0.1	0.461	(−0.01 to 0.01)
Obsessive compulsive	−0.1	0.226	(−0.01 to 0.00)
Interpersonal sensitivity	−0.3	**0.003**	(−0.02 to −0.01)
Global severity index	−0.0	0.651	(−0.02 to 0.01)

*Adjusted for confounding factors; age, gender, smoking and BMI. HDL dependent variable, symptom clusters independent variables.

Bold values are statistical significant numbers.

## Discussion

The main finding of this study was a negative association between serum HDL and phobic anxiety, paranoid ideation, anger hostility and interpersonal sensitivity symptom clusters from SCL-90-R. These findings remained significant after adjusting for the most known confounding factors including BMI, age, gender, smoking, HbA1c, and diabetes. No associations were found between TG, LDL and psychiatric symptoms.

A reduction in HDL has been described in other studies on psychiatric disorders and was inversely associated with cognitive deficits, mood disorders and schizophrenia (21, 32). One study reports HDL levels to be lower in depressed patients who did not receive any treatment in comparison to those who already receive treatment (33). Another study suggested lower serum HDL to be a predictive biomarker for severity of depression in first episode drug-naïve depressed patients, although the sample size was low (n=15) (34). Moreover, low serum HDL level is suggested to be associated with long-term depressive symptomatology, as depressed patients with symptom duration of more than 3 years have been demonstrated to have lower serum levels of HDL than patients with symptom duration of less than 3 years (22). Further, melancholic and atypical features of depression were independently associated with lower HDL and higher LDL levels (25). HDL has been described lower also in schizophrenia and bipolar disorders compared to a healthy control group (20).

Interpersonal sensitivity is defined as a stable trait characterized by ongoing concerns about negative social evaluation. Significantly inverse association with HDL and interpersonal sensitivity has been reported, in line with our results (35, 36).

Lower levels of HDL have also been significantly associated with symptoms of aggression and violent behaviour (37). Anger and hostility are symptoms seen in several psychiatric conditions, including mood disorders, anxiety and psychoses (38). The risk of developing a cardiovascular disorder is increased in those who have low HDL levels (36). Previous studies report that HDL is decreased across many psychiatric diagnoses. Patients with depression and aggressive behaviour have shown to be at risk of cardiovascular disorders, and treatment of depression may have a conceivable effect on the reduction of this risk (36, 39).

In contrast to our findings regarding TG and LDL, a recent study reports significant association between low TG and LDL levels and depression (40). While other studies revealed increased TG in major depressive and anxiety disorders, and a positive relationship with depression severity (41, 42). Furthermore, in a study on 30 patients with different anxiety disorders, borderline-high or high lipid levels (cholesterol, LDL/HDL ratio) were found almost 3 times as often as in control patients (43). Triglyceride levels were significantly increased in patients with first episode psychosis, while LDL levels were reduced in a metanalysis conducted (44). In a study on healthy men demonstrating hostility and angry affect behaviour, a relationship with elevated TG and LDL was described (45). Results concerning TG and LDL levels in psychiatric disorders are so far inconsistent.

Taken together, we did not find any significant association between TG, LDL and general psychiatric symptoms in this study. However, HDL was significantly inversely associated with the intensity of four of the symptom clusters. The fact that these associations remained significant after adjusting for lifestyle-related factors, especially BMI, diabetes and smoking, suggests that these associations are not secondary to lifestyle-related factors. This could imply that it is the HDL profile, rather than the cholesterol levels, that has a role in general psychiatric disorders, and this is compatible with one other study (36).

Several explanations are suggested to understand the pathophysiology. Lipids may influence psychiatric disorders by altering signalling pathways, serotonin metabolism and the HPA axis (40). Dysfunctional HPA axis activity, serotonin release as well as lipid metabolism may potentially contribute to the severity of depression (40). There are indications that HDL also acts through the inflammatory pathway and HDL has a systemic anti-inflammatory effect (22). Thus, lipids may act though many complex and systemic concomitant pathways.

Altered levels of lipids in relation to psychiatric disorders suggest a role of lipids in brain cell function, a potential psychopathological mechanism serving as a potential target for prevention and treatment of psychiatric disorders. However, the findings also shed light on the cardiovascular and metabolic comorbidities in psychiatric disorders. Patients with severe psychiatric disorders may have unfavourable lipid profiles (8, 46), and a 15–20 years shorter life expectancy than the healthy population. The increased morbidity and mortality have been ascribed in part to lifestyle and side effects of medications. Depression can also influence appetite potentially leading to low serum TG and LDL. Some publications suggest a common origin for metabolic and psychiatric disorders (47, 48). A genetic basis for the causal relations between metabolic syndrome and psychiatric disorders has been suggested (49). There are arguments that psychiatric disorders should be regarded neuro-endocrine-immuno-metabolic, and lipids having a role in the pathogenesis (46, 50).

In line with an effect of lipids on psychiatric symptoms, studies on statins reducing TG and LDL and increasing HDL have been reported to have a positive effect on symptoms of anhedonia, psychomotor retardation, anxiety and sleep disturbances in depression (51). Moreover, statins as add-on treatment to SSRI antidepressants have been demonstrated to have antidepressant effect in patients diagnosed with depression (52). Findings are however not conclusive, underscoring the need for further research.

The strengths of this study include use of a well-known and validated symptom scale, the SCL-90-R, and adjustment for the most known potential confounding factors. One novelty is the assessment of symptoms rather than diagnoses, also across diagnostic categories (53). All clinical assessments and interviews in this study were performed by the first author, thus eliminating possible inter-rater variability (54). The laboratory used trained technicians with a standardized protocol for the biochemistry analyses. Blood samples were taken fasting in the morning, eliminating diurnal variation (55).

The limitations of this study, apart from the cross-sectional design without a control group and single-centre sampling, is the inability to consider other possible confounding factors that can be related to lipids and lipid metabolism. Possible influence of dietary habits and physical exercise factors, blood pressure, waist circumference and other physical conditions as well as medication were not adjusted for and represent potential uncontrolled variables. However, none of the patients enrolled in this study had severe or acute physical illnesses. Further, measurements of peripheral lipids may not necessarily reflect the brain status, but there is evidence from animal studies that peripheral levels may reflect the brain levels by transport across the blood brain barrier. Cholesterol lowering statins in guinea pigs showed reduction in brain 24S-Hydroxycholesterol levels, the transportable form of cholesterol (56). The cerebrospinal fluid in humans contains a large number of lipoproteins resembling HDL and proteins probably involved in immune responses and inflammation (57). In addition, HDL may also cross the blood brain barrier and affect the brain steroidogenesis (58).

The use of plasma or serum sampling for lipid parameters have been reported to result in 3–5% variation in cholesterol measures. Values for cholesterol and TG were higher for serum than for plasma, while values for HDL were not different (44, 59). The sample size in this study is relatively small (n= 132) and could potentially influence the result. However, other similar studies in this field have smaller numbers (n <100) (37, 45, 60, 61).

## Conclusion

In conclusion, these data suggest a possible role of HDL in the association of psychiatric disorders across general psychiatric diagnoses and cardiovascular risk. New therapies directed at lipid metabolism may have potential for preventing or treating relevant subgroups or symptoms in psychiatric disorders and mitigating cardiovascular risk.

## Data Availability

The raw data supporting the conclusions of this article will be made available by the authors, without undue reservation. Requests to access the datasets should be directed to hedda.beate.soloy-nilsen@nordlandssykehuset.no.
